# Machine Learning models for the Identification of Cognitive Tasks using Autonomic Reactions from Heart Rate Variability and Electrodermal Activity

**DOI:** 10.3390/bs9040045

**Published:** 2019-04-25

**Authors:** Hugo F. Posada-Quintero, Jeffrey B. Bolkhovsky

**Affiliations:** 1 Department of Biomedical Engineering, University of Connecticut, Storrs CT 06269, USA; 2 Naval Submarine Medical Research Laboratory, Groton CT 06340, USA; jeffrey.b.bolkhovsky.civ@mail.mil

**Keywords:** heart rate variability, electrodermal activity, autonomic nervous system, psychomotor vigilance task, working memory, ship search

## Abstract

Indices of heart rate variability (HRV) and electrodermal activity (EDA), in conjunction with machine learning models, were used to identify one of three tasks a subject is performing based on autonomic response elicited by the specific task. Using non-invasive measures to identify the task performed by a subject can help to provide individual monitoring and guidance to avoid the consequences of reduced performance due to fatigue or other stressors. In the present study, sixteen subjects were enrolled to undergo three tasks: The psychomotor vigilance task (PVT), an auditory working memory task (the n-back paradigm), and a visual search (ship search, SS). Electrocardiogram (ECG) (for HRV analysis) and EDA data were collected during the tests. For task-classification, we tested four machine learning classification tools: k-nearest neighbor classifier (KNN), support vector machines (SVM), decision trees, and discriminant analysis (DA). Leave-one-subject-out cross-validation was used to evaluate the performance of the constructed models to prevent overfitting. The most accurate models were the KNN (66%), linear SVM (62%), and linear DA (62%). The results of this study showed that it is possible to identify the task a subject is performing based on the subject’s autonomic reactions (from HRV and EDA). This information can be used to monitor individuals within a larger group to assist in reducing errors caused by uncoordinated or poor performance by allowing for automated tracking of and communication between individuals.

## 1. Introduction

Professions require individuals to perform tasks that demand different skills and present various physiological challenges, including fast reaction to visual or auditory commands, working memory, visual search, or staying awake for extended periods of time, among others. Each type of task produces stress that elicits an autonomic reaction [1]. However, the stress produced by different tasks has different components, resulting in task-type specific autonomic responses [2,3]. Furthermore, the risks that represent the reduction in performance, due to various stressors, vary among tasks [4,5]. Using the specific effects of a given task on the sympathetic and vagal (parasympathetic) branches of the autonomic nervous system (ANS) to identify the task being performed will help to deploy strategies to foster human performance and minimize the risk and economic burden that the misperforming of different tasks represents [6,7]. 

In this study, we measured ANS response to three different tasks: Psychomotor vigilance task, auditory working memory, and continuous visual search. These tasks, along with a baseline measurement, allow for the analysis of variable requirements of vigilance as well as the analysis of variable stimulus rates. Task differences based on these factors have shown to have multiple effects on the ANS [8,9]. The psychomotor vigilance task (PVT) measures performance through reaction time of a concurrently presented stimulus. The PVT allows for the observation of short periods of non-action caused by the deterioration of vigilant attention [10]. Furthermore, measures derived from the PVT exhibit a demonstrated correlation with both the circadian rhythm and the lack of sleep [11]. The PVT was included as a vigilance-based task with a high stimulus rate. We used the n-back paradigm to assess working memory [12]. The n-back paradigm describes a task in which people are presented with a stimulus and must respond with a positive or negative response on whether that stimulus was equivalent to one that was previously presented. Working memory suffers deficits with prolonged wakefulness [13]. The n-back acted as a high-stimulus rate and non-vigilance-based task. The final task type, continuous visual search, is expected to elicit distinct physiological responses as compared to the first two task types used, and functions as a vigilance-based and low stimulus rate task [14]. 

The dynamics of the ANS have been largely studied using the spectral analysis of heart rate variability (HRV) and electrodermal activity (EDA) [15]. Both HRV and EDA can be measured non-invasively and can differentiate between sympathetic and parasympathetic control. EDA, which measures the changes in electrical conductance of the skin (based on sweat production), has recently gained popularity in response to the need for techniques providing better results than HRV for the separate assessment of the branches of the ANS [16,17,18]. EDA is considered a purely-sympathetic measure, because parasympathetic nerves do not innervate the eccrine sweat glands [19].

Some studies have performed task classification based on measures of brain activity and indices of HRV [20,21,22,23,24]. Those studies generally found high within-task (i.e., multi-level) classification accuracy (>80%), but low cross-task classification accuracy (around 45%). To the best of our knowledge, this paper describes the first study examining the feasibility of using indices of ANS based on EDA and HRV to classify cognitive tasks. In particular, this study seeks to explore classification models based on the differences and similarities in the effects that tasks involving psychomotor vigilance, auditory working memory, and continuous visual search have in the ANS. It is hypothesized that physiological variables obtained from HRV and EDA will expose the difference in autonomic reaction produced by the three different tasks. We performed three steps of analysis: First, we looked for changes in the indices of HRV and EDA between the three tasks using a repeated-measurements analysis, to assess how sensitive those indices are to the differences in the tasks; second, we trained different models to classify the different tasks using the HRV and EDA indices, to determine the best model and the most useful indices for each task; and third, we evaluated the performance of the best models in the trials performed by the subjects during a 24-h period of wakefulness.

## 2. Materials and Methods

### 2.1. Subjects

Sixteen healthy volunteers (8 males, 8 females; 18 to 45 years old) were recruited for this study. Within two hours of waking up on the day the study initiated, participants came to the experimental facility located at the Storrs campus of the University of Connecticut. During the first hour after arrival, participants were instructed on how to perform the three tasks of the experiment and performed a training trial. After the training trial, each subject performed a trial of the experimental protocol (described below) every other hour, completing a total of 12 trials during the 24-h period, trial 12 occurring after 25 h. Participants remained in the building for approximately 25 h to allow for the completion of all trials. The study protocol was approved by the Institutional Review Board of the University of Connecticut, in compliance with all applicable Federal regulations governing the protection of human subjects. All subjects gave written informed consent in accordance with the Declaration of Helsinki.

Five minutes before each task trial, participants were instructed to place a set of three Ag/AgCl electrodes on their chest for recording electrocardiographic (ECG) signals. Stainless steel electrodes placed on the index and middle fingers of each participant’s non-dominant hand were used to collect EDA signals. An HP 78354A ECG monitor (Hewlett-Packard) was used to collect ECG, and a galvanic skin response amplifier FE116 (ADINSTRUMENTS) was used to collect EDA. No filtering was used during the signal recording. The EDA device was calibrated to zero before every trial. 

### 2.2. Protocol

As stated previously, the experiment consisted of three tasks: (1) The PVT, (2) the n-back task, and (3) the ship search (SS) task, always performed in the same order. Prior to the three tasks, baseline ECG and EDA measurements were collected by having participants remain stationary for four minutes. Subjects were not aware that the difference between the tasks would be measured.

*Psychomotor Vigilance Task.* For the PVT, participants were asked to click the left button of the mouse as quickly as possible after they saw a number appear on the screen. The numbers appeared at randomly generated intervals between 2 and 10 s. Participants performed the PVT task using an available piece of software installed on a desktop computer [25]. The same computer was used for all subjects. The PVT task took 10 min to complete.

*N-back test.* The n-back test is designed to challenge the participants’ working memory. It lasted about 10 min. During this task, participants sat in front of computer with two speakers facing toward them. Tones with different lengths and pitches were played as stimuli to the participants. For each stimulus, participants were asked to determine if the stimulus was the same as, or different from, the previously played stimulus. Participants pressed pre-determined keys to respond positively (stimuli were identical) or negatively (stimuli were different). 

**Figure 1:**
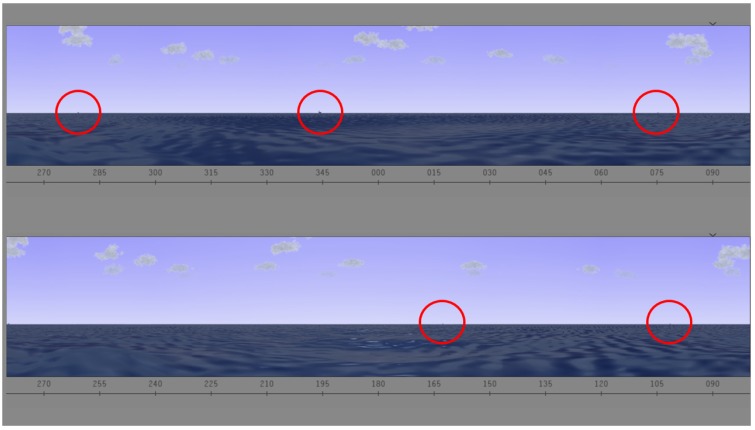
Ship search task. There are five ships in the screen.

*Ship Search.* In this task, participants performed a continuous visual search. This task took 20 min. Participants were asked to sit in front of a computer screen. Participants were provided a search task, illustrated in Figure 1, in which they were asked to identify when and where ships appeared on an interactive screen. The visual was designed to simulate the view of the water from a submarine periscope. Throughout this task, ships appeared on screen at different times and subjects were instructed to report when they spotted them by pressing the space bar and to verbally express the specific location based on the system of coordinates available. 

### 2.3. HRV and EDA Indices

Measures of autonomic nervous system (ANS) activation based on HRV and EDA data were computed. Subjects were required to maintain their torso (where ECG was collected) and non-dominant hand (where EDA electrodes were connected) still while performing the trials, to secure quality of physiological data. 

#### 2.3.1. Indices of HRV

For HRV analysis, four minutes of clean ECG were extracted from the beginning of baseline, PVT, n-back, and SS task data. Noise and motion artifacts were removed from the ECG signals using a band-pass filter (0.05–40 Hz). The R peaks were detected using a previously validated algorithm [26,27]. After automatic R peaks were detected, segments were manually inspected to ensure that no beat was missed. The R–R interval time series was transformed to an evenly time-sampled signal by cubic spline interpolation (sampling frequency = 4 Hz). A Blackman window (length of 256 points) was applied to each segment, and the fast Fourier transform was calculated for each windowed segment. Finally, the power spectra of the segments were averaged. 

We computed the indices of low frequencies of HRV (HRVLF [ms^2^], 0.045 to 0.15 Hz), high frequencies of HRV(HRVHF [ms^2^], 0.15 to 0.4 Hz), and the indices normalized to the total power of HRV (HRVLFn, HRVHFn, in normalized units) [15]. Indices obtained from the LF of HRV (HRVLF and HRVLFn) are referred as indices of sympathetic control, and indices from the HF power (HRVHF and HRVHFn) are regularly used as indices of parasympathetic control.

#### 2.3.2. Indices of Electrodermal Activity

EDA signals were processed in the time domain to obtain the skin conductance level (SCL, [µS]) and skin conductance responses (SCRs) [16]. The SCL represents the low-frequency shifts and SCRs represent the rapid phase shifts of the EDA signal. Indices based on spectral analyses of EDA (time-invariant and time-variant) have been reported recently as more sensitive and consistent measures of sympathetic control [28,29]. In this study, the first two minutes of EDA data were extracted from baseline and the three tasks, to compute the indices of EDA in the time and frequency domains. The SCL and the SCRs were obtained [16]. SCL is defined as the mean value of the tonic component of the EDA, and the SCRs are the phasic changes of the EDA signal. Based on the detected SCRs, the frequency of non-specific SCRs (NS.SCRs) was computed as the count of SCRs whose maximum value is higher than a predefined threshold (0.05 µS, in this study), per minute [16]. SCL and NS.SCRs were extracted using the SparsEDA, a feature extraction scheme that is based on a non-negative sparse deconvolution [30]. 

The power spectral index of EDA (EDASymp [µS^2^]) was computed by integrating the power in the range sensitive to cognitive stress as defined in a previous study (0.045 to 0.25 Hz) [28]. The spectra of EDA were calculated using Welch’s periodogram method with 50% data overlap. A Blackman window (length of 128 points) was applied to each segment, the fast Fourier transform was calculated for each windowed segment, and the power spectra of the segments were averaged. To obtain the time-varying index of EDA (TVSymp, normalized units), the time-varying spectral representation of EDA was computed using variable frequency complex demodulation [31]. The instantaneous amplitude of components within the frequency power previously defined (0.08 to 0.24 Hz) were used to compute TVSymp [29]. 

### 2.4. Statistics

The physiological indices of ANS collected in this study are: SCL, NSSCRs, EDASymp, TVSymp, HRVLF, HRVLFn, HRVHF, and HRVHFn. The three analyses performed in this study were: Repeated measurements analysis between the tasks, task-classification analysis, and performance of the classifiers for subsequent measurements in a 24-h wakefulness period. First, we conducted repeated measurements analysis to evaluate the differences in the HRV and EDA indices between the tests, including baseline. Normality of the EDA and HRV indices was tested using the one-sample Kolmogorov–Smirnov test [32,33,34]. As all indices met the normality criteria, we performed the one-way analysis of variance (ANOVA) to test for significant differences between tasks. We used the Bonferroni method for correction of multiple comparisons.

For the task-classification analysis, four approaches were tested: k-nearest neighbor classifier (KNN, k = 1) [35], support vector machines (SVM) [36], decision trees [37], and discriminant analysis [38,39]. In SVM, linear (LSVM) and gaussian (GSVM; C = 1, γ = 2.6) kernels were used. In the discriminant analysis, linear, quadratic, and Mahalanobis distances quadratic approaches were used. These classifiers were used to evaluate all the possible combinations of the eight indices of HRV and EDA to discriminate between the three tasks and baseline, using the data from the first time the subjects performed the tasks. Leave-one-subject-out cross-validation was used to evaluate the performance of the machine learning models and preventing overfitting. Accuracy was computed as the number of correct classifications into the four groups (baseline, PVT, n-back and SS), divided by the total number of classifications performed. Finally, data were analyzed to see if there was any change in the accuracy of the classification as a function of time, by using the data collected in the subsequent eleven trials of the test. Data processing and analysis were performed in MATLAB.

## 3. Results

**Figure 2:**
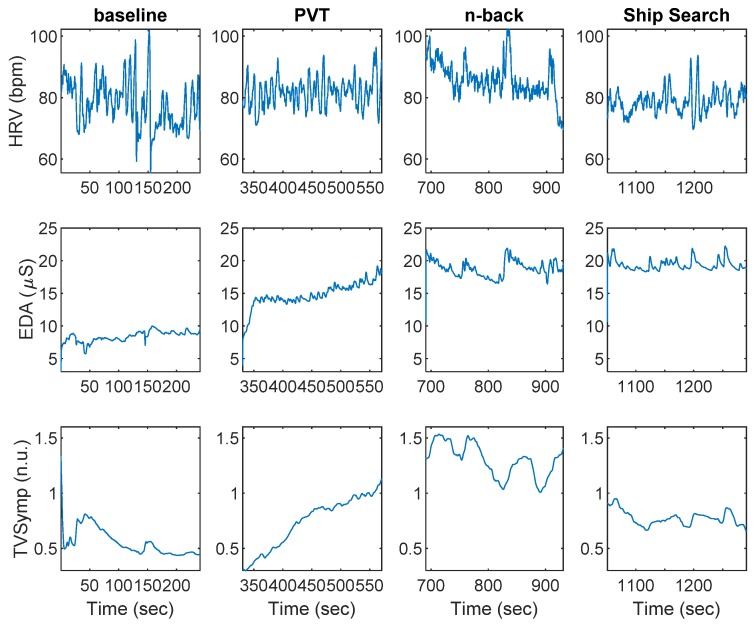
Heart rate variability (HRV) and electrodermal activity (EDA) data collected during baseline, the psychomotor vigilance task (PVT), n-back, and ship search for a given subject.

Figure 2 shows an example of HRV and EDA data collected during baseline, PVT, n-back, and SS, for a given participant. Indices of HRV and EDA obtained during the baseline, PVT, n-back, and SS tasks are shown in Table 1. The SCL was the only index to exhibit differences between PVT and baseline. The SCL, EDASymp, HRVLF, HRVLFn, and HRVHF indices were significantly different between n-back and baseline. The NS.SCRs, HRVLFn, HRVHF, and HRVHFn indices were significantly different between n-back and PVT. The SCL, EDASymp, TVSymp, and HRVHF were different between SS and baseline. Only the HRVHF exhibited significant differences between SS and PVT. The TVSymp, EDASymp, and HRVLFn were significantly different between SS and n-back.

Table 2 includes the resulting accuracy and indices required for the best classification model for each machine learning approach. The highest accuracy was achieved by the KNN (66%), LSVM (62%), and linear discriminant analysis (LDA) classifiers (62%). The lowest overall accuracy was exhibited by the quadratic discriminant analysis (QDA) model. HRVLFn was the only index present in the most accurate combination for each model. As for the indices of EDA, SCL was present in all the models, except QDA. Remarkably, all models required at least one index of EDA and one index of HRV.

Testing all the possible combinations of the eight indices of HRV and EDA available, the set of features that provided the highest accuracy for the KNN classifier was: SCL, EDASymp, TVSymp, HRVLFn and HRVHF. The models achieved the maximum accuracy using between two and five indices. M-QDA used only two indices to achieve the maximum accuracy of 53% (SCL and HRVLFn). Table 3 includes the confusion matrices for the KNN and LSVM models, for the leave-one-subject-out cross-validation analysis. The baseline data were correctly classified 69% and 63% of the times, respectively. PVT was assigned correctly 69% with the KNN model and 56% of the times with the LSVM. The n-back was assigned correctly 56% of the times in the KNN and 69% of the times with the LSVM. Finally, SS was correctly classified 69% and 63% of the times, respectively.

**Figure 3:**
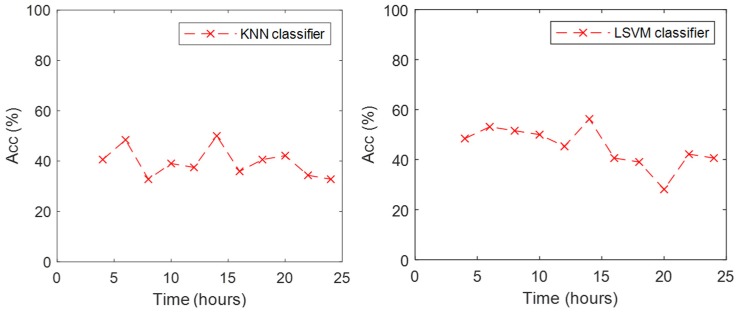
Accuracy of KNN (**left**) and LSVM (**right**) classifiers during the eleven trials.

As KNN and LSVM exhibited higher performance than the other classifiers, we analyzed what happened to the accuracy of those classifiers with the data from the subsequent eleven trials while the participants were kept awake. Figure 3 includes the accuracy achieved by these two classifiers for the eleven trials. Models were trained with the full data set of the first trial. The KNN classifier presented an accuracy around 40% for the trials before 20 h, and a minimum of 33% was observed at 24 h. Accuracy increased to 50% at 14 h. The LSVM classifier presented an accuracy around 50% for the trials before 18 h, a minimum of 28% at 20 h. Its accuracy increased to 42% in the trial at 22 h but was reduced to 40% for the last trial at 24 h. In general, the LSVM classifier exhibited higher accuracy in the data collected after the training trial.

## 4. Discussion

We can summarize the main findings of this study with the following: (1) We found that psychomotor vigilance, working memory, and continuous visual search tasks had different effects in the ANS, noticeable in HRV and EDA indices; (2) the three tasks and baseline were classified with an overall accuracy of up to 66%; (3) the indices with more significant differences between tasks were the SCL, TVSymp, and HRVLFn, which are markers of sympathetic tone [15,16,29]; (4) considering the machine learning models overall, the indices that provided most information for classifying the tasks were the HRVLFn and SCL.

The activation of the sympathetic system is the natural response to coordinate the adaptive reactions of the organism [1]. Combining the sympathetic indices with others like HRVHF, which is mostly sensitive to the parasympathetic function [15], allowed for higher accuracy in classification of the tasks. The classification models were useful in exploring the effects of simple tasks in the ANS, and how those effects change over time as subjects are awake for a prolonged period. 

The SCL was significantly different between all three tasks and the baseline, but not between the tasks. This index was significantly increased from baseline, a stage in which no task was performed, to the active stage. The SCL is also referred to as the tonic component, and is related to several non-sympathetic activity factors but also to the level of attention and reactiveness of the subject to simple tasks [40,41]. None of the other indices exhibited a significant increase from PVT to baseline. As SCL has been linked to reactiveness, this observation suggests that such a task required only a significant attention and reactiveness to the participants. The other indices of EDA (NS.SCRs, EDASymp, and TVSymp) are related to the phasic (high-frequency) components, which are evoked by central or peripheral mechanisms [42] and are known to be linked to attention and stimulus novelty [43]. These phasic-component indices, and the indices of sympathetic tone derived from HRV (HRVLF and HRVLFn), exhibited lower values during n-back compared to other tasks (including baseline). This suggests that n-back stimuli (auditory) represented lower novelty to the participants compared to PVT and SS, although it still required a similar level of attention (no differences in SCL).

Significantly higher values observed in indices of sympathetic control based on EDA (tonic and phasic) were found between SS and baseline. The only index that exhibited differences between SS and PVT was HRVHF, a marker of parasympathetic control. This suggests that the only difference between the psychomotor and the visual search was a higher parasympathetic tone. We can summarize the findings of the repeated-measurements analysis as follows: All tasks required a significant level of attention, compared to the baseline period; n-back task stimuli represented lower novelty and required similar attention compared to other tasks, and lower parasympathetic tone compared to PVT; SS task stimuli presented higher novelty than baseline and n-back and elicited a lower parasympathetic tone compared to PVT.

Overall, the KNN and LSVM classifiers exhibited the best performance, based on the highest achieved accuracy of the leave-one-subject-out cross validation approach. In the subsequent eleven trials, LSVM exhibited a higher classification accuracy. Those trials were performed under increased wakefulness of the subject, which made it a more challenging task for the classifiers. For this reason, an accuracy of about 50% for four classes is acceptable because it means it captured differences in the effects of the tasks on the ANS.

PVT (a psychomotor task) and n-back (auditory working memory) were highly distinguishable tasks. The KNN model assigned only 6% of the PVT samples as n-back and 19% of the n-back samples as PVT. The LSVM misclassified 6% of the time for both tasks. Similarly, baseline and the SS task were accurately classified for both models, as KNN misclassified 6% of the time between those two tasks, and LSVM assigned 13% of the SS samples to baseline and 0% of the baseline (BL) samples to the SS class. BL and PVT seemed to be the most similar classes, as the LSVM model misclassified the BL and PVT classes 25% of the time.

KNN and LSVM exhibited a reduction in accuracy on the classification between 20 and 22 h. Previous studies have found a reduction in performance at 22 h of wakefulness [11,44,45]. The low accuracy suggests that the activation of the ANS was diminished (or at least different) after 20 h of wakefulness, compared to the trials in normal conditions (first trials). This suggests the conclusion that the inappropriate activation of the ANS in sleep-deprived subjects is causative of the reduced performance. By the end of the 24 h, the performance exhibited a recovery, which suggests the ANS is re-activated. This has also been observed in the recovering performance reported in previous studies and is thought to be related to the circadian rhythm [11,44,45].

These findings support the classification of human performance based on tasks with variable stimulus response rates and vigilance requirements. Specifically, these methodologies could lead to the development of systems that track actions and behavior over time. By identifying the task a person could face at any specific point in time, it becomes possible to assist in coordinating operations done by large groups through tracking changes in individual activity and adapting to provide support to those that need it. For example, these methods could be utilized during larger scale field operations, such as those frequently performed by the military.

## 5. Conclusions

We studied the effects of three different tasks on human autonomic response. Subjects performed the PVT, n-back, and SS task every two hours during a 24-h period. For the first time, we have combined measures of autonomic response based on HRV and EDA as input features for machine learning classification models, which allowed us to classify individual activity using non-invasive measures with a relatively high accuracy. Traditional measures of EDA, along with the novel techniques that incorporate spectral information to the analysis of the signal, contribute to the improved accuracy of the task classification. Furthermore, HRV and EDA techniques are much easier to deploy (for instance, in a wearable device) than those for assessing brain activity (e.g., electroencephalogram). 

The results of this study can help to understand and identify, from a physiological point of view, the type of task a person is performing. This methodology could allow the deployment of task-specific risk management strategies with the ability to track individuals within larger groups. 

## Figures and Tables

**Table 1:** Indices of HRV and EDA during baseline, PVT, n-back, and ship search (SS) tasks.

Indices	BL	PVT	n-back	SS
SCL	0.19 ± 3.9	3.5 ± 2.4 *	4.6 ± 2.5 *	5.6 ± 2.5 *
NS.SCRs	2.1 ± 0.93	3.1 ± 1.1	2.1 ± 1.1 ^†^	2.5 ± 1.1
EDASymp	0.079 ± 0.1	0.12 ± 0.1	0.062 ± 0.064	0.19 ± 0.12 * ^‡^
TVSymp	0.29 ± 0.21	0.44 ± 0.2	0.28 ± 0.15	0.49 ± 0.25 * ^‡^
HRVLF	11 ± 3.6	10 ± 3.5	7.2 ± 3 *	9 ± 2.1
HRVLFn	0.35 ± 0.093	0.4 ± 0.082	0.24 ± 0.085 * ^†^	0.38 ± 0.088 ^‡^
HRVHF	6.5 ± 1.5	6.5 ± 1.4	4.9 ± 1.6 * ^†^	4.9 ± 1.3 * ^†^
HRVHFn	0.21 ± 0.069	0.26 ± 0.08	0.16 ± 0.061 ^†^	0.2 ± 0.083

* significant differences to baseline, ^†^ significant differences to PVT, ^‡^ significant differences to n-back. SCL, skin conductance level; NS.SCRs, non-specific skin conductance responses; EDASymp, sympathetic component of the EDA; TVSymp, time-varying index of sympathetic tone; HRVLF, low-frequency components of heart rate variability (HRV); HRVLFn, normalized low-frequency components of HRV; HRVHF, high-frequency components of HRV; HRVHFn, normalized high-frequency components of HRV.

**Table 2:** Maximum leave-one-subject-out cross-validation accuracy for each task-classification machine learning model.

Model	Accuracy	Indices
KNN	66%	SCL, EDASymp, TVSymp, HRVLFn, HRVHF
LSVM	62%	SCL, NSSCR, EDASymp, HRVLFn, HRVHF
GSVM	56%	SCL, NSSCR, HRVLFn
LDA	62%	SCL, NSSCR, HRVLFn, HRVHF
QDA	52%	NSSCR, HRVLF, HRVLFn, HRVHFn
M-QDA	53%	SCL, HRVLFn

KNN, k-nearest neighbor classifier; LSVM, linear support vector machines; GSVM, gaussian support vector machines; LDA, linear discriminant analysis; QDA, quadratic discriminant analysis; M-QDA, Mahalanobis quadratic discriminant analysis; SCL, skin conductance level; NS.SCRs, non-specific skin conductance responses; EDASymp, sympathetic component of the EDA; TVSymp, time-varying index of sympathetic tone; HRVLF, low-frequency components of heart rate variability (HRV); HRVLFn, normalized low-frequency components of HRV; HRVHF, high-frequency components of HRV; HRVHFn, normalized high-frequency components of HRV.

**Table 3:** Confusion matrices for the KNN and LSVM classifiers.

KNN						LSVM				
	BL	PVT	n-back	SS			BL	PVT	n-back	SS
BL	69%	13%	13%	6%		BL	63%	25%	13%	0%
PVT	13%	69%	6%	13%		PVT	25%	56%	6%	13%
n-back	19%	19%	56%	6%		n-back	13%	6%	69%	13%
SS	6%	25%	0%	69%		SS	13%	19%	6%	63%

KNN, k-nearest neighbor; LSVM, linear support vector machine; BL, baseline; PVT, psychomotor vigilance task; SS, ship search.
